# Priming effect of root-applied silicon on the enhancement of induced resistance to the root-knot nematode *Meloidogyne graminicola* in rice

**DOI:** 10.1186/s12870-018-1266-9

**Published:** 2018-03-27

**Authors:** Li-Ping Zhan, De-Liang Peng, Xu-Li Wang, Ling-An Kong, Huan Peng, Shi-Ming Liu, Ying Liu, Wen-Kun Huang

**Affiliations:** State Key Laboratory for Biology of Plant Diseases and Insect Pests, Institute of Plant Protection, Chinese Academy of Agricultural Sciences, 100193 Beijing, People’s Republic of China

**Keywords:** Silicon, *Meloidogyne graminicola*, Ethylene pathway, Lignin, Callose, Induced defense

## Abstract

**Background:**

Silicon (Si) can confer plant resistance to both abiotic and biotic stress. In the present study, the priming effect of Si on rice (*Oryza sativa* cv Nipponbare) against the root-knot nematode *Meloidogyne graminicola* and its histochemical and molecular impact on plant defense mechanisms were evaluated.

**Results:**

Si amendment significantly reduced nematodes in rice roots and delayed their development, while no obvious negative effect on giant cells was observed. Increased resistance in rice was correlated with higher transcript levels of defense-related genes (*OsERF*1, *OsEIN*2 and *OsACS*1) in the ethylene (ET) pathway. Si amendment significantly reduced nematode numbers in rice plants with enhanced ET signaling but had no effect in plants deficient in ET signaling, indicating that the priming effects of Si were dependent on the ET pathway. A higher deposition of callose and accumulation of phenolic compounds were observed in rice roots after nematode attack in Si-amended plants than in the controls.

**Conclusion:**

These findings indicate that the priming effect may partially depend on the production of phenolic compounds and hydrogen peroxide. Further research is required to model the ethylene signal transduction pathway that occurs in the Si-plant-nematode interaction system and gain a better understanding of Si-induced defense in rice.

## Background

Rice (*Oryza sativa*) is an important staple food crop for the majority of the human population and is a model organism for monocotyledonous plants [35, 36]. The most damaging nematodes attacking this crop are the root knot nematode *Meloidogyne graminicola*, the root rot nematode *Hirschmanniella oryzae* and the cyst nematode *Heterodera* spp. [5]. To date, *M. graminicola*is the most important of the three and is prevalent in the major rice-producing countries of the world [1, 14, 28]. *M. graminicola* can induce substantial growth retardation in rice seedlings and causes up to 80% yield loss of aerobic rice, lowland and deep-water rice in Southeast Asia and other regions [5, 14, 37]. Crop rotation with dicots and a fallow season may reduce nematode populations and improve rice yield [44]. Chemical nematicides (carbofuran, phorate, chlorpyriphos) have been used as seed treatments or soil applications for the management of *M. graminicola* in fields and have achieved significant suppression of galling, egg mass production and soil populations of this nematode [26, 41]. Many antagonistic bacteria and fungi (*Pseudomonas fluorescens, Paecilomyces lilacinus*) were reported to promote plant growth and produce substances that inhibit nematode egg hatch or kill nematodes [42]. However, the potential negative impact of chemical nematicides on the environment and humans has led to a total ban on or restricted use of these chemicals. The disadvantages of crop rotation, such as the requirement for increased expertise and specialized equipment and differing management practices, limit its application. With increased interest among growers in environmentally friendly control methods and in the reduction of chemical nematicide usage, induced resistance (IR) has been proposed as a new management strategy for this destructive nematode in recent years.

IR is a physical state of enhanced defensive capacity elicited by natural or synthetic agents that activate plant defense systems against specific biotic stresses (e.g., fungi, bacteria, viruses and nematodes) or abiotic stresses (e.g.*,* salt, temperature, drought and chemicals) [10, 12, 48]. The plant defense system consists of preexisting physical and chemical barriers as well as inducible defense responses, which are activated after pathogen infection [22]. The synthesis of phytoalexins, strengthening of cell walls, and production of antifungal proteins were observed in different plants after pathogen infection. Salicylic acid (SA), jasmonic acid (JA), ethylene (ET) and other signaling pathways were found to play key roles in the regulation of defense responses in different plants [16, 47].

The role of silicon(Si) in conferring plant resistance to both abiotic and biotic stress has received increased attention [7, 11, 30, 40, 53]. Several studies have demonstrated that soil-applied Si can induce systemic defenses against different pathogens in many plants. Amendment of cucumber plants with soluble Si resulted in a marked stimulation of chitinase activity and rapid activation of peroxidases after infection with *Pythium* spp. [9]. Root-applied Si significantly increased the activity of phenylalanine ammonia-lyase in cucumber and decreased the powdery mildew disease index [30]. The addition of Si to the nutrient solution significantly reduced both lesion size and area under the blast progress curve in rice [7]. Amendment of plants with calcium silicate remarkably reduced the number of root galls and eggs of various species of *Meloidogyne* in bean, tomato and coffee [13]. The addition of calcium silicate to Si-deficient soil significantly decreased the number of galls and eggs of *M. exigua* and increased the concentration of lignin-thioglycolic acid derivatives of coffee [43]. According to Guimarães et al. [19], potassium silicate was effective in reducing the number of nematode eggs of *M. incognita* in sugar cane. Si treatment adversely affected *M. javanica* development in soybean, common bean and rice and reduced nematode penetration of rice roots [32]. However, no Si treatment influenced the development and penetration of nematodes in maize. To date, the mechanisms by which Si provides protection against plant nematodes have yet to be fully elucidated. Some authors propose that Si forms a physical barrier in cell walls, preventing the penetration of fungal hyphae or nematode stylets into host tissues [40]. Others believe that Si may be associated with an increase in plant resistance to pathogens through the production of phenol-like compounds; in the levels of phytoalexins; in an increase in the activity of peroxidase (POX), polyphenol oxidase (PPO) and phenylalanine (PAL); and through the strong activation of pathogenesis-related (PR) genes [19, 30, 43]. In those experiments, however, the effect of Si on the suppression of subsequent disease was evaluated only in inoculated leaves or roots without evaluating the whole plant. In addition, no in-depth studies have been performed on the effects of amended Si on defense-related genes in rice. Therefore, it is still unknown whether Si amendment can induce defense reactions against *M. graminicola* infection.

This paper analyses the priming effect of root-applied Si on rice to defense against *M. graminicola*, and the defense mechanism involved in the induced resistance of rice. The results presented here demonstrate that Si can reduce nematode infection in rice. The ET pathway and the production of phenolic compounds and hydrogen peroxide in rice roots may be involved in this phenomenon.

## Results

### Si has no toxic effect on the behavior of nematodes

**Fig. 1:**
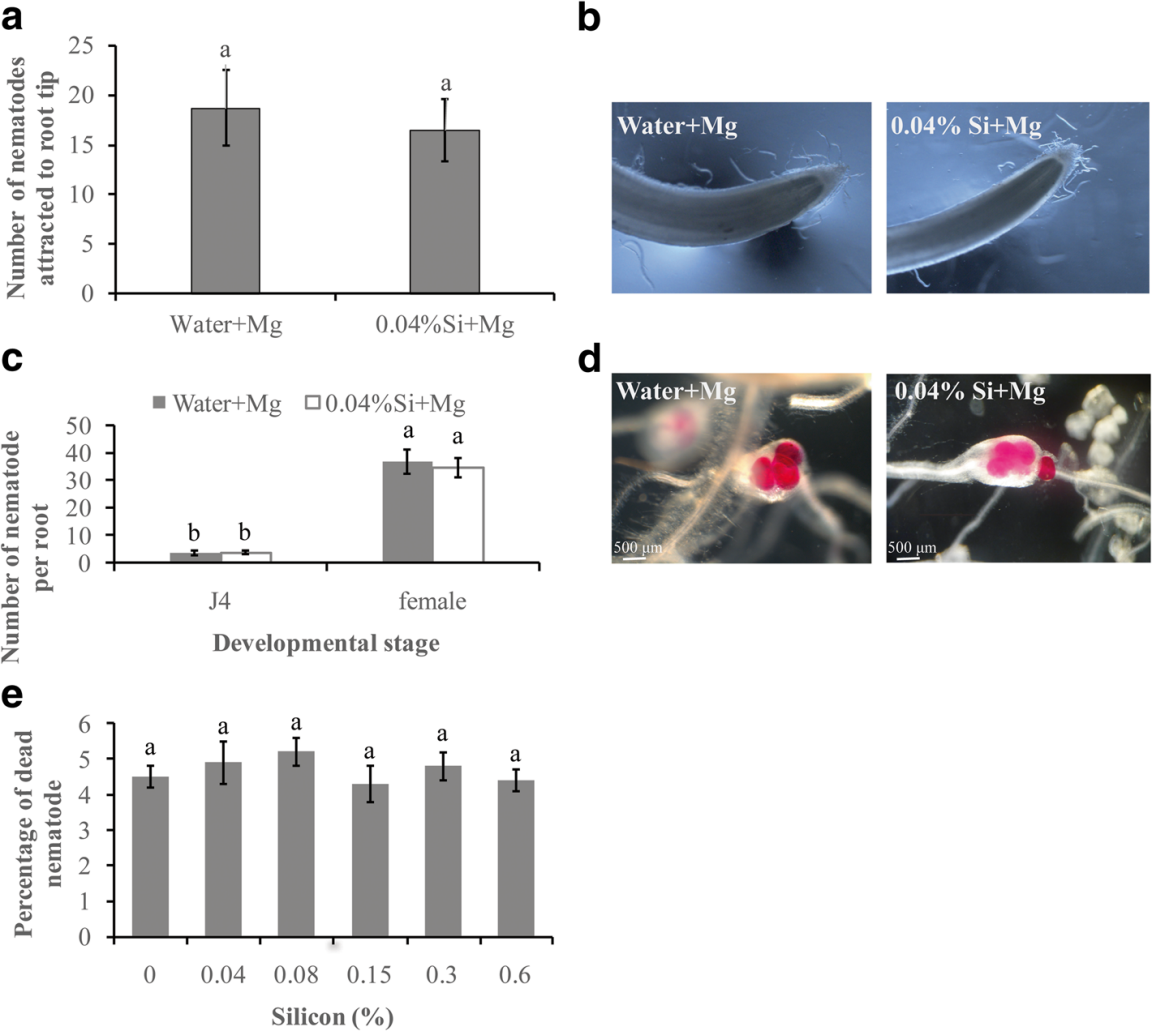
Direct effect of silicon soaking on *Meloidogyne graminicola* (Mg). **a** Nematodes attracted within 5 mm from the root tip were counted at 6 h after inoculation. **b** Attraction of *M. graminicola* towards the rice root tip after drenching with 0.04% Si or water. **c** Infectivity and development of silicon-incubated (Si) and water-incubated Mg in rice roots. **d** Mg nematodes in silicon-incubated (Si) and water-incubated plants were counted and photographed at 14 dpi. **e** The percentage of dead juveniles after incubation for 72 h in various concentrations of silicon and water. The experiment was repeated three times, with 6 individual plants in each replicate. Data are presented as the mean ± SE of six replicates

To determine whether Si has a direct effect on the attractiveness of roots to *M. graminicola,* we examined the number of attracted nematodes around the root tips treated with Si or water. At 6 h post inoculation (hpi), nematodes attracted to the Si-treated root tips (16.5 ± 3.1) was not significantly different from those of the control root tips (18.7 ± 3.8) (Fig. 1a and b). This result indicates that the tested Si exudates do not prevent the attraction of rice root to *M. graminicola*.

To determine whether Si has a direct effect on the infectivity of nematodes, the penetration and development of nematodes pre-treated with Si or water inoculated on rice roots were monitored. At 14 days post inoculation (dpi), most of the nematodes had developed into fourth stage (J4) and females. No significant difference was observed in the number of J4 or adult females between the Si-incubated and water-incubated groups (Fig. 1c and d). These data show that incubation of nematodes in Si neither inhibited the penetration nor delayed the development of *M. graminicola* inside the rice roots.

To further evaluate whether Si has a negative effect on nematode behavior, *M. graminicola* was incubated in different concentrations of Si for 72 h. No significant differences in nematode mortality were observed between the Si solutions (4.7 ± 0.4) and water (4.5 ± 0.3) at doses ranging from 0.04% to 0.6% (Fig. 1e). These data suggest that the investigated doses of Si have no nematicidal effect on *M. graminicola* up to 72 h.

### Si amendment induces rice defense against *M. graminicola*

**Fig. 2:**
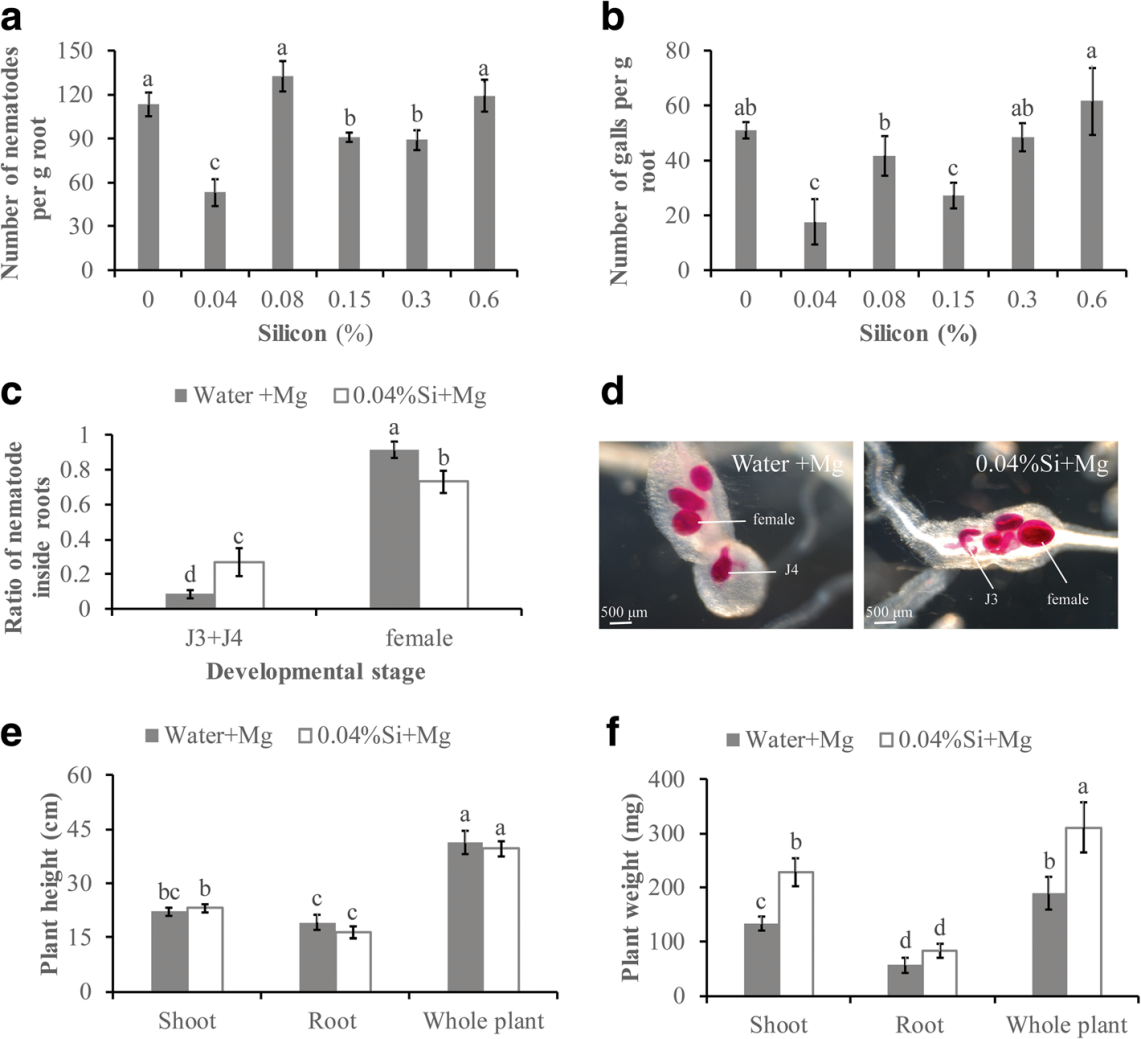
Effect of silicon amendment on plant growth and nematode infection. **a** Nematodes per gram of silicon-amended and non-amended Nipponbare roots were counted at 14 dpi. **b** Root galls per gram of silicon-amended and non-amended Nipponbare roots were counted at 14 dpi. **c** The ratio of nematodes in rice roots at different developmental stage is the value of the number of nematodes in different life stage (female or J3/J4) divided by the total number of nematodes in roots. **d** Roots were stained with acid fuchsin and photographed. Female and J3/J4 were outlined with white lines, respectively. **e** Plant height at 14 dpi. **f** Plant weight at 14 dpi. The bars in the different graphs represent the mean ± SE of data from three independent biological replicates, each containing 6 individual plants. Different letters indicate statistically significant differences (Duncan’s multiple range test at *P* ≤ 0.05)

To evaluate the potential priming effect of Si, different concentrations of Si were amended to the plant growth substrate and plant infection rates were evaluated at 14 dpi in a preliminary experiment. Based on the observation that amendment with 0.04% Si is the most effective dosage in inducing the defense of rice against *M. graminicola*, further studies were performed with this concentration. Amendment with 0.04% Si resulted in a significant reduction of nematodes (53.1 ± 7.8%) at 14 dpi (Fig. 2a). Since the J3/J4 nematodes would have initiated gall formation, Si amendment also resulted in a significant reduction of root galls (65.5 ± 3.1%) at 14 dpi (Fig. 2b). Thereafter, the result implies that plant defense is playing a role in the reduced galling and nematode after Si-treatment. In addition, the development of nematodes was slightly delayed in Si-treated plants compared with that in non-treated control plants. At 14 dpi, the ratio of adult females in 0.04% Si-treated plants (0.73 ± 0.06) was significantly lower than that of non-treated plants (0.92 ± 0.08), whereas a higher ratio of third- and fourth-stage juveniles (J3 + J4 s) (0.27 ± 0.04) was observed in Si-treated roots than in non-treated roots (0.08 ± 0.05) (Fig. 2c and d). Measurements of the height and fresh weight of the rice plants demonstrated that the Si amendment did not restrict plant growth. No significant difference was observed in plant height between Si-treated and non-treated plants (Fig. 2e). The weight of shoots in Si-treated nematode-inoculated plants was significantly higher than that of non-treated inoculated plants (Fig. 2f), but no significant difference in root weight was observed. These data suggest that Si amendment can induce rice defense against *M. graminicola*, which can alleviate nematode-induced plant growth reduction. Si decreases the infection of *M. graminicola* and delays nematode development inside roots without restraining plant growth.

**Fig. 3:**
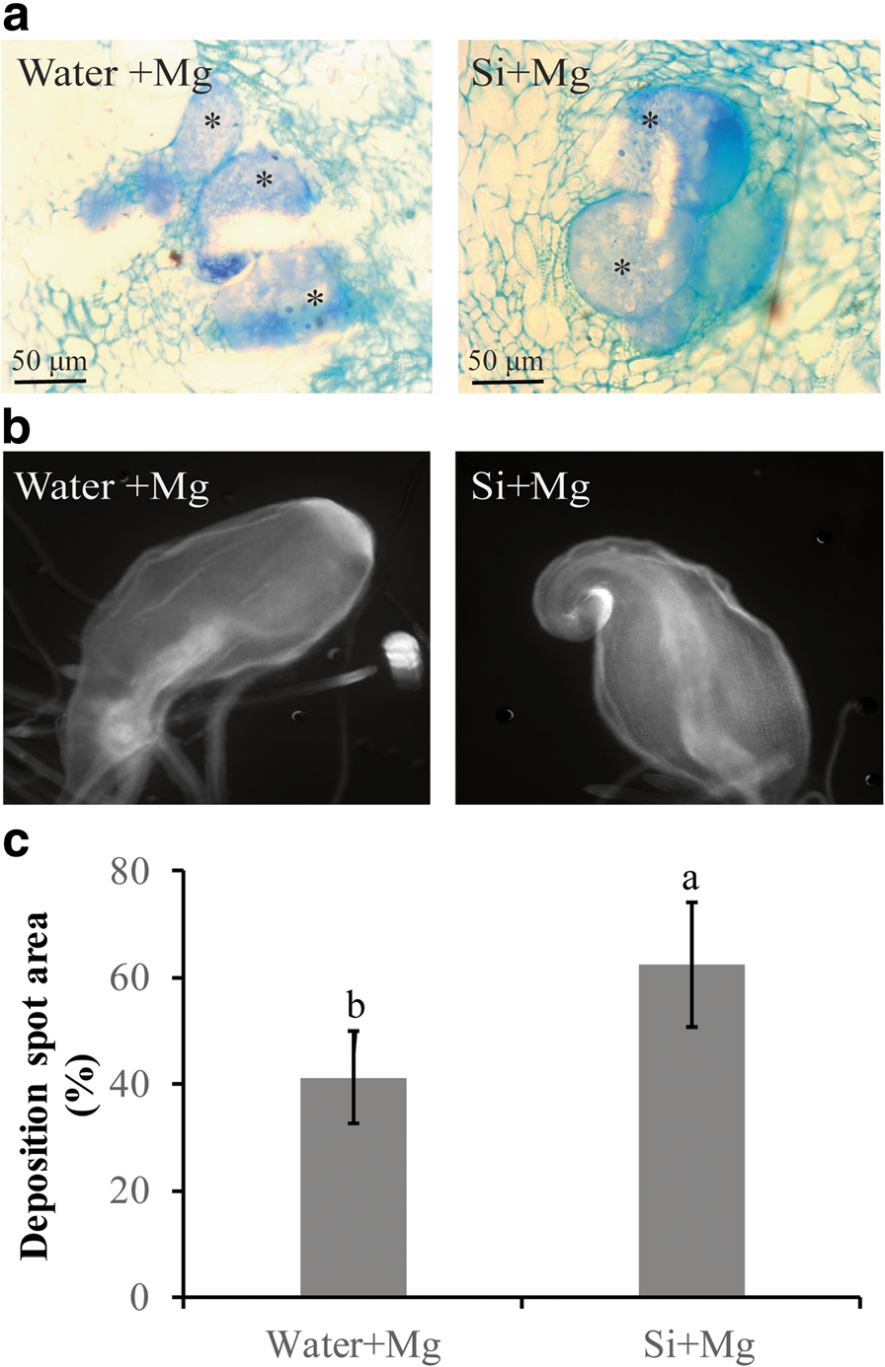
Analysis of callose and giant cell structure in root galls after Si treatment. **a** Giant cells (*) in silicon-amended rice root galls and control root galls were stained at 7dpi with toluidine blue solution and observed by microscopy. **b** Callose deposition in root galls was examined under UV light at 7 dpi. **c** Quantification of callose deposition was performed at 7 dpi using ImageJ software. Data presented are the mean ± SE of two independent experiments, each performed using ten galls

A microscopic investigation of feeding sites revealed no significant difference in the quantitative characteristics of giant cells in the Si-amended roots versus the non-amended roots. The number of giant cells in the Si-amended roots was similar to that in the non-amended roots (Fig. 3a). These data demonstrate that Si-amendment has no visible negative effect on giant cell development.

### Si primes basal defense against nematode infection

To further investigate whether the addition of Si activated the basal defense of rice, the presence of callose, hydrogen peroxide (H_2_O_2_) and lignin was investigated in nematode-induced gall tissues. Although the prominence and density of callose spots in Si-amended galls was not significantly higher than in non-amended galls at 7 dpi (Fig. 3b), the average area of the callose deposition spot in the galls of Si-amended plants increased by 21.3% compared to that of non-amended plants (Fig. 3c). These data show that callose deposition in root galls may be one of the drivers of Si-induced resistance in rice plants.

**Fig. 4:**
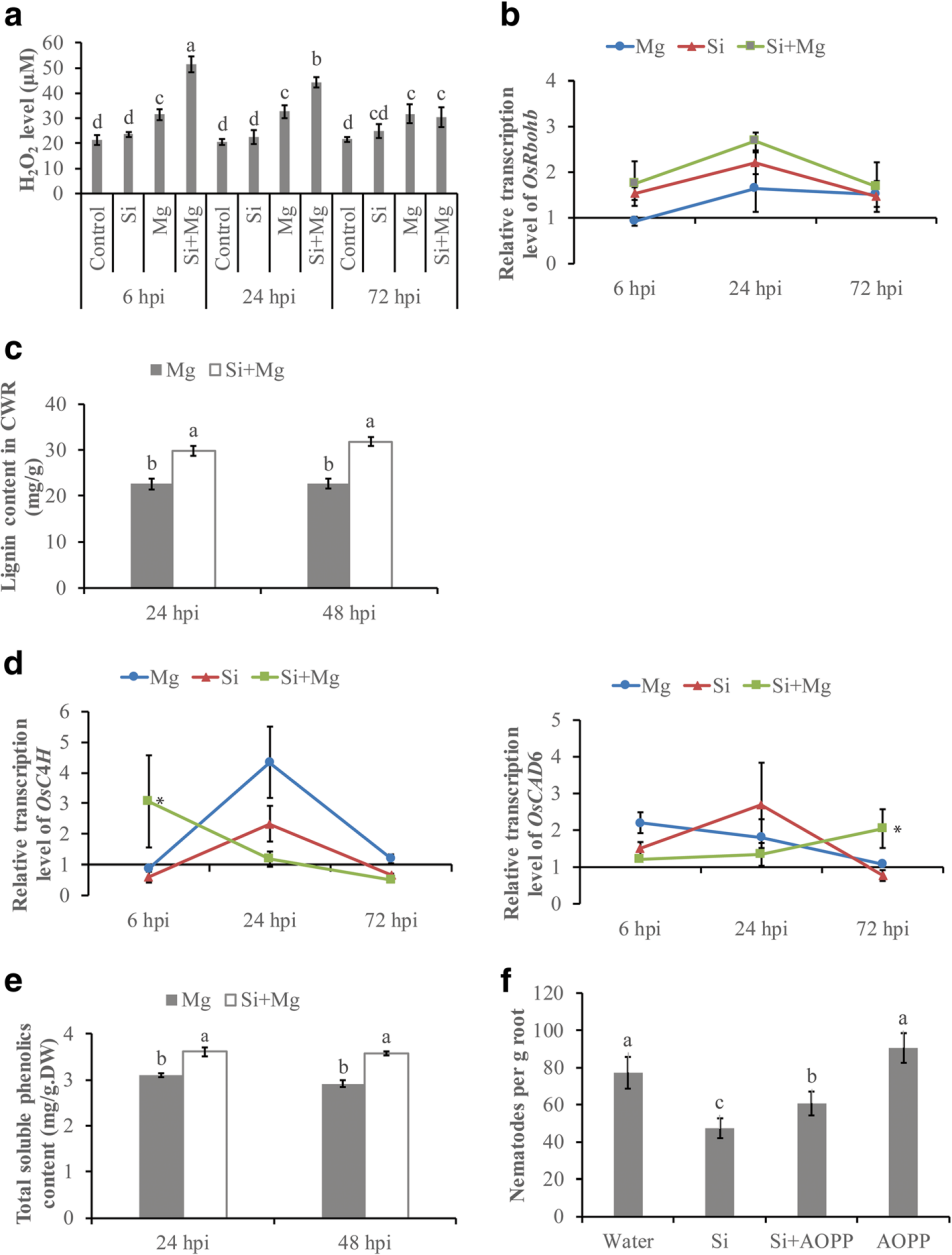
Analysis of H_2_O_2_, phenolics and lignin after Si treatment. **a** The H_2_O_2_ content per gram of roots was measured using an UV spectrophotometer at 390 nm. The bars represent the mean ± SE of four replicates, each containing a pool of six roots. Different letters indicate significant differences (Duncan’s multiple range test with *P* ≤ 0.05). **b** Quantitative RT-PCR analysis of *OsRbohB* gene related to H_2_O_2_production. **c** The lignin content in the cell wall residue (CWR) of rice roots was determined at 280 nm using the AcBr assay. **d** Quantitative RT-PCR analysis of *OsC*4*H* and *OsCAD*6 genes in the phenylpropanoid pathway. Relative transcript levels of *OsC*4*H* and *OsCAD*6were analyzed at 6, 24 and 72 h post-inoculation and normalized to three internal reference genes, *OsEXP*, *OsEif*5*C* and *OsEXPnarsai*. Data are shown as relative transcript levels in comparison with the control roots (expression level set at 1). The bars represent the mean expression levels±SE from two independent biological replicates and three technical replicates, each containing a pool of 6 plants. Asterisks indicate significant differential expression (Duncan’s multiple range test with P ≤ 0.05). **e** Total soluble phenolics content in roots of dried weight(DW) was measured using an UV spectrophotometer at 725 nm. Measurements were conducted at 24, 48 h post-inoculation after silicon amendment (Si + Mg) or water treatment (Mg). The bars represent the mean ± SE of the lignin content of 6 plants. Different letters indicate significant differences. **f** Effect of L-2-aminooxy-3-phenylpropionic acid (AOPP, an inhibitor of phenylpropanoid biosynthesis) on nematode infection. AOPP (100 μM) was applied to 2-week-old rice roots with or without 0.04% silicon 1 day before inoculation. Nematodes in the roots were counted at 14 days post-inoculation. The bars represent the means of the data from two independent biological replicates, each containing six plants. Different letters indicate significant differences (Duncan’s multiple range test with P ≤ 0.05)

H_2_O_2_ performs multiple important functions in the early defense responses of plants [54]. To investigate whether Si is capable of generating reactive oxygen species (ROS) in the rice-*M. graminicola* interaction, the H_2_O_2_ level of rice roots was analyzed at different time points. Si alone did not trigger an H_2_O_2_ response in rice roots as no significant differences in H_2_O_2_ levels were observed at 72 hpi after Si treatment compared with control roots. However, on nematode challenge, Si-treated plants showed induced H_2_O_2_ accumulation compared with non-treated plants by 140.8% ± 15.1% at 6 hpi and 116.7% ± 9.8% at 24 hpi, but no significant difference in H_2_O_2_ level was observed between Si-treated alone and Si treated inoculated plants at 72 hpi (Fig. 4a). In addition, the expression level of *OsRboh*B, a H_2_O_2_ synthesis gene known to be involved in the plant immune response [55], was investigated using qRT-PCR. After Si treatment, although the *OsRboh*B gene showed the highest expression in Si-treated inoculated treatment at all tested time, no significant difference was detected in Si-treated inoculated treatment compared to other treatments (*P* > 0.05) (Fig. 4b). These data suggest that Si amendment may activate the rapid generation of ROS to induce defense against the infection of root-knot nematodes.

Lignification confers mechanical strength to plant cell walls to enhance host defense against pathogen invasion [3, 39, 52]. A higher level of lignin was observed at 24 and 48 hpi in Si-amended inoculated plants than in non-amended inoculated plants (Fig. 4c), indicating that cell wall lignification can be enhanced by Si amendment. Transcript analysis of *OsC*4*H* and *OsCAD*6, members of the phenylpropanoid biosynthesis pathway, further confirmed this result. Enhanced transcription levels of *OsC*4*H* and *OsCAD*6 were observed in Si-treated plants compared to those in untreated controls at 6 and 24 hpi, respectively (Fig. 4d). Soluble phenolics can be incorporated into lignin precursors, and the lignification of the cell walls can prevent host penetration by pathogens. Higher levels of soluble phenolics were also observed in Si-amended plants at the two tested time points after inoculation (Fig. 4e). To further investigate the role of the phenylpropanoid pathway in Si-induced defense against *M. graminicola* in rice, an inhibitor of phenylpropanoid biosynthesis, L-2-aminooxy-3- phenylpropionic acid (AOPP), was applied to rice plants at 24 h before nematode inoculation. AOPP treatment enhanced nematode infection and reduced the effect of Si-mediated defense against *M. graminicola* (Fig. 4f). These results indicate that the biosynthesis of phenylpropanoids is involved in Si-induced defense against *M. graminicola* infection and that lignin fortification of cell walls can be induced by Si amendment.

### Si-induced defense against *M. graminicola* is mediated by the ET pathway

**Fig. 5:**
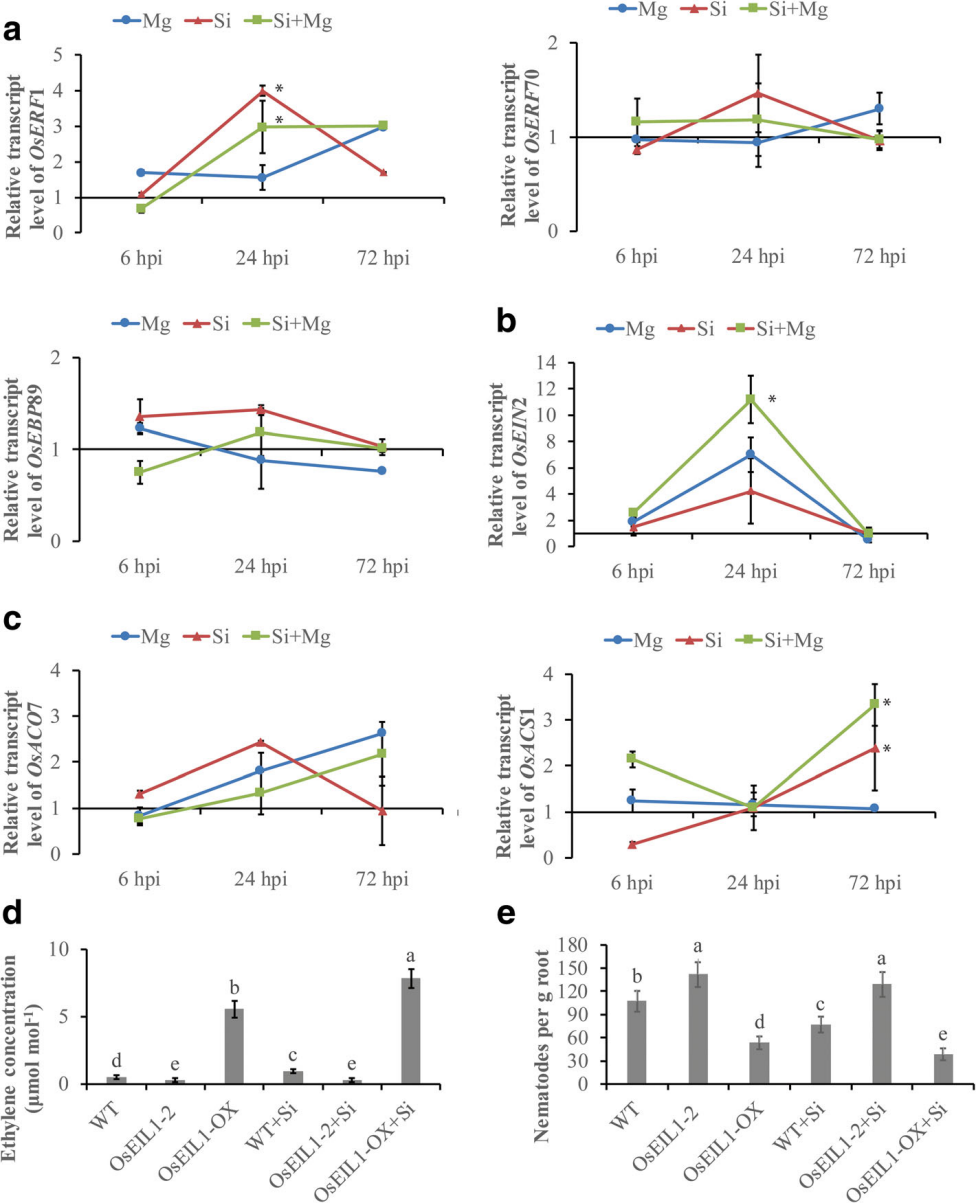
Quantitative RT-PCR analysis of genes in the ethylene pathway. **a** Relative transcript levels of the ET-response genes *OsERF*1, *OsERF*70 and *OsEBP*89. **b** Relative transcript levels of the ET-signaling gene *OsEIN*2. **c** Relative transcript levels of the ET-biosynthesis genes *OsACS*1 and *OsACO*7. Gene expression levels were analyzed at 6, 24 and 72 h post-inoculation and normalized with three internal reference genes, *OsEXP*, *OsEif*5*C* and *OsEXPnarsai*. Data are shown as relative transcript levels in comparison with the control roots (expression level set at 1). The bars represent the mean expression levels±SE from two independent biological replicates and three technical replicates, each containing a pool of 6 plants. Asterisks indicate significant differential expression (Duncan’s multiple range test with P ≤ 0.05). **d** Ethylene concentration in rice plants. The ethylene insensitive3-like1 gene, *OsEIL1–2-*RNAi line deficient in ET signaling and the transgenic line overexpressing *OsEIL1-OX* amended with or without silicon were transplanted in a sealed plexiglass bottle and inoculated with 100 J2 s each. Samples of air in each plexiglass box were analyzed with a gas chromatograph at 24 hpi. **e** Effect of silicon amendment on nematode infection in ethylene mutants. The ethylene insensitive3-like1gene, *OsEIL1–2-*RNAi line deficient in ET signaling and the transgenic line overexpressing *OsEIL1-OX* amended with or without silicon were inoculated with 100 J2 s each, and nematodes in rice roots were investigated at 14 dpi. A *japonica* wild type (WD), *Taijing*394, was used as a control. The bars represent the means of the data from two similar experiments, each containing 10 individual plants. Different letters indicate significant differences (Duncan’s multiple range test with P ≤ 0.05)

Ethylene is involved in mediating plant responses to various biotic and abiotic stresses [25]. To investigate whether the ET pathway participates in Si-induced defense against RKNs in rice, the expression levels of genes involved in ET biosynthesis (*OsACS*1, *OsACO*7), ET signaling (*OsEIN*2) and ET response (*OsERF*70, *OsERF1*, *OsEBP*89) were analyzed at different time points. Transcription of the ET response gene *OsERF*1 was significantly up-regulated in Si-amended versus non-amended plants at 24 hpi (Fig. 5a). Transcription of the ET signaling gene *OsEIN*2 was increased 24 hpi following Si-treatment compared to that in the untreated control (Fig. 5b). No significant differences in the expression of *OsACO*7, an ET-biosynthesis gene, was observed at any tested time. A significant enhancement of *OsACS*1expression was observed in Si-treated plants at 72 hpi compared to the expression level in the untreated control (Fig. 5c).

*OsEIL1* is involved in the ethylene signal transduction pathway and acts as a positive regulator of the ethylene response in rice [31]. To further investigate the role of the ET response in Si-induced defense against *M. graminicola*, ethylene level and nematode infection in two transgenic plants, *OsEIL1–2-*RNAi*,* which causes deficiencies in ET signaling, and *OsEIL1-OX*, which increases ET signaling by 15- to 20-fold [56], were investigated at different time points. Ethylene concentration was higher in Si-treated *OsEIL1-OX* plants than in the untreated plants at 24 hpi (Fig. 5d). Similar results were also observed in wild type plants. However, no significant difference was observed in Si-treated *OsEIL1–2-*RNAi plants than in untreated plants at tested time point. At 14dpi, more nematodes were observed in the *OsEIL1–2-*RNAi plants than in the wild-type plants (Fig. 5e). However, no significant difference was observed between Si-treated and untreated *OsEIL1–2-*RNAi plants. A significantly lower number of nematodes was observed in the transgenic line *OsEIL1-OX* than in the wild-type plants. Fewer nematodes were observed in *OsEIL1-OX* transgenic plants after Si treatment. Therefore, the priming effect of silicon was only observed in the overexpressed transgenic lines but not in the insensitive lines. These results indicate that the ET signaling pathway is involved in Si-induced defense against *M. graminicola* in rice.

## Discussion

In the present study, we have demonstrated that amendment of rice roots with Si reduces infection by the RKN *M. graminicola*. This Si-induced defense is associated with the priming of a multifaceted defense response, including the enhancement of ET-pathway expression, deposition of callose, and the accumulation of H_2_O_2_ and phenolic compounds. Priming is a unique physiological state induced by the application of low levels of so-called ‘priming agents’, which enables plant cells respond to a challenge of pathogen invasion in a more rapid and robust manner than non-primed cells [10]. Our results show that 0.04% Si amendment resulted in least number of root galls than that of 0.08% or higher doses. In our previous research, 1.2% biochar amendment resulted in less nematodes in rice roots than that of 5% biochar amendment [20]. Ji et al. [24] found that the priming induced by low level of β-aminobutyric acid (BABA) is capable of inducing rapid and effective defense responses to root-knot nematodes.

In compatible interaction between plants and nematodes, nematode infection elicits a set of localized responses around the infected host cells [18]. These responses include oxidative burst, changes in cell wall composition and activation of the expression of pathogenesis-related genes. Many of these responses are regulated by cross-communicating signal-transduction pathways, within which plant hormones fulfill central roles. H_2_O_2_ has been shown to inhibit the viability of diverse microbial pathogens, and its oxidative potential contributes to plant wall strengthening during plant-pathogen interactions [54]. Our research showed that a significant accumulation of H_2_O_2_ was observed in the Si-amended roots at early stages, indicating a priming effect mediated by Si on the nematode-induced oxidative burst. A previous study showed that increased production of H_2_O_2_ leads to polymerization of monolignols by peroxidase and subsequent lignin formation [4]. Our results showed that significantly higher levels of lignin and soluble phenolics were present in Si-amended plants than in non-amended plants. Si application has been associated with an increase in the density of silicified buliform cells in the leaf epidermis that help to increase resistance against blast disease in rice [27]. According to Rodrigues et al. [39], Si-mediated resistance to rice blast disease was related to the accumulation of phenolic compounds. Rodrigues et al. [39] also found that a differential accumulation of glucanase, peroxidases, and PR-1 transcripts was associated with a higher concentration of phenolic compounds and lignin in rice plants supplied with Si. During the plant defense response, lignin or lignin-like phenolic compound accumulation in cell walls was found to occur in a variety of plant-microbe interactions and is, thus, considered a first line of defense against successful penetration by invasive pathogens [3].

RKNs have the capacity to penetrate the cell wall with their stylet and gain access to cellular nutrients [29]. However, nematodes face many barriers of early post-invasive defense, including cell wall lignification and the rapid accumulation of ROS followed by changes in metabolic and hormonal profiles [3, 21]. Callose is a component of cell wall-associated structures at particular stages of growth and differentiation. Callose deposition can be induced by wounding, infection by pathogens and other physiological stresses [46]. Our results show that silicon amendment enhances callose deposition in rice plants after nematode infection. Ji et al. [24] also observed that the defense-activating molecule β-aminobutyric acid (BABA) induced a significant increase in callose deposition in rice roots after infection by RKN. In *Arabidopsis*, enhancement of callose deposition in the syncytia enhanced its defense against the cyst nematode *H. schachtii* [2]. However, in our previous study, biochar amendment did not induce callose deposition in root galls after RKN inoculation [20]. These results indicate that defense mechanisms in plants can vary due to different priming agents.

Plants require a broad range of defense mechanisms to effectively combat microorganism invasions, including preexisting physical and chemical barriers, synthesis of phytoalexins, enhanced strengthening of cell walls, and the production of antifungal proteins [47]. Previous studies showed that the plant hormone ethylene plays a key role in the regulation of defense responses in many plants. Spokas et al. [45] observed that exogenous ET production from biochar-amended soil induced resistance in plants to different pathogens. Fudali et al. [15] showed that ET-overproducing Arabidopsis plants are less attractive to *M. hapla*. Nahar et al. [34] observed that exogenous ET supply on shoots induced a strong systemic defense response of rice and the JA pathway played a pivotal role in ET-induced defense. However, exogenous brassinosteroids were found to be able to suppress rice defense against root-knot nematodes through with the JA pathway [35]. The present study shows that ET is a key player in Si-induced systemic defense against root-knot nematode in rice. The ET response gene *OsERF*1 was significantly induced not only in Si-amended non-infected plants compared to non-amended non-infected plants but also in Si-amended infected plants compared to non-amended infected plants. The enhanced transcription of ET response genes may contribute to the defense of rice plants and reduce nematode infection in Si-treated plants. In addition, Si had no effect on the insensitive line *OsEIL1–2*-RNAi, whereas a significantly suppressed response was observed in the transgenic line *OsEIL1-OX,* further confirming ET’s role in the regulation of defense responses of rice against RKNs. Similar results were obtained in our previous study on biochar-induced resistance in rice. Exogenous biochar application increased the expression of the ET-response genes *OsERF*1 and *OsEBP*89 in rice and reduced infection by the RKN *M. graminicola* [20]. These results indicate that the effects of Si on rice plants are dependent on ET signaling.

## Conclusion

We demonstrated that Si amendment in rice roots potentiates a priming defense response against the RKN *M. graminicola*. This Si-induced resistance is associated with the activation of the ET response, deposition of callose, accumulation of H_2_O_2_ and phenolic compounds after nematode attack. Further research is required to model the ethylene signal transduction pathway that is induced in the Si-plant-nematode system and to gain a better understanding of Si-induced defense in rice.

## Methods

### Plant materials and silicon

Soluble silicon fertilizer (SiO_2_ ≥ 99.8%, granule diameter 10–20 nm, pH 4–6) was obtained from Zhongke Qirun Biological Organic Fertilizer Company, Hengshui China, and stored in sealed plastic containers until use. Synthetic absorbent polymer (SAP) substrates were prepared with a 1:400 (w: v) mixture of sand and SAP [21, 38]. The silicon-fertilizer was mixed with SAP substrate in different concentrations (0, 0.04, 0.08, 0.15, 0.3, and 0.6%) before sowing.

Rice seeds (*Oryza sativa* cv. Nipponbare) were obtained from the US Department of Agriculture (GSOR-100) and cultivated in Hanshou County, Hunan Province, China. One transgenic *OsEIL1–2-*RNAi line deficient in ET signaling and a transgenic *EIL* overexpressing (15- to 20-fold) line *OsEIL1-OX* were constructed as Yang et al. [56] and cultivated in our experimental base of Langfang city, Hebei Province. Rice seeds were soaked in 5.25% sodium hypochlorite for 5-10 min and germinated at 28 °C for 4 d. One geminated seed was sown in each polyvinyl-chloride (PVC) tube containing SAP. Rice seedlings were maintained in a greenhouse at 26 °C with 70–75% relative humidity and a 16 h/8 h light/dark regime and irrigated with 20 ml of Hoagland solution twice per week.

### Nematode extraction and culture

*M. graminicola* was collected from fields in Hanshou County, Hunan Province and maintained on Nipponbare in a greenhouse at 26–28 °C. One month after inoculation, nematode eggs were separated from the root galls under a microscope and hatched at 26 °C for 48 h. Second-stage juvenile (J2) suspensions were filtered through 25-μm sieves and resuspended in distilled water with a concentration of approximately 100 nematodes per milliliter [6].

### Direct effects of Si on nematode behavior

Si fertilizer was dissolved in distilled water at concentrations of 0.04, 0.08, 0.15, 0.3 and 0.6% (w: v) for 24 h. The suspensions were centrifuged at 10,000 g for 3 min, and the supernatant was collected to analyze the toxicity of Si to the nematodes. Approximately 100 J2 s were placed in a 6-well culture plate (3.5 cm diameter) containing 1 ml of Si solution or distilled water. After 72 h, 1 N NaOH was added to the solution to count the dead/live nematodes under a stereomicroscope [8]. The experiment was performed three times with six replicates each.

To test the direct effect of Si on the attraction of *M. graminicola* to rice root, 23 g Pluronic F-127 powder (Sigma-Aldrich, China) was dissolved in 100 mL of sterile water at 4 °C [21]. Roots of 2-week-old plants were drenched with 20 ml of 0.04% Si exudates or water. Twenty-four hours later, a 1-cm-long root tip was cut and placed into a culture plate containing approximately 100 J2 s and 1 mL Pluronic gel. Nematodes attracted within 5 mm from the root tip were counted at 6 hpi. The experiment was performed three times with six replicates each.

To determine the direct effect of Si on the infectivity of nematodes, *M. graminicola* J2 s were incubated in Si solution for 48 h before inoculation. Control nematodes were incubated in distilled water. Two-week-old rice plants were inoculated in the roots with 100 J2 s and maintained in the greenhouse as described above. At 14 dpi, root samples were washed until clear and dipped in 0.6% NaOCl for 5 min. Root samples were wrapped separately with a microcloth and stained with acid fuchsin for 3 min as described in Nahar et al. [34]. After destaining in 4% acidified glycerol for 3–4 days, nematodes inside the roots in each developmental stage were counted under a stereomicroscope. To calculate the ratio of nematodes, the number of nematodes in different life stage (female or J3/J4) was divided by the total number of nematodes in roots using Microsoft Excel 6.0 (Redmond, Washington, USA).

### Si-induced resistance against *M. graminicola*

To test whether Si addition induced rice defense against *M. graminicola*, each 2-week-old plant that was maintained in the appropriate concentration of Si was inoculated with approximately 100 J2 s. At 14 dpi, the plant height and fresh weight were measured. Then, root galls were counted and root samples were stained as described in Nahar et al. [34]. Nematodes inside roots and their developmental stages were counted. The experiment was performed three times with six replicates each.

To obtain a more detailed understanding of the role of the ET response in Si-induced defenses against RKNs, an *OsEIL1–2*-RNAi line deficient in ET signaling and a transgenic line overexpressing *OsEIL1-OX* [56] were treated with Si or water and inoculated with 100 J2 s. A *japonica* wild type, *Taijing*394, was used as a control. At 24 hpi, ethylene concentrations in rice plants were analyzed as Gil et al. [17] with minor modifications. Briefly, rice plants were transplanted in a plexiglass bottle. The bottles were hermetically sealed with plastic tape after inoculation with nematodes. A sample of air in each plexiglass box was extracted with a 1-ml syringe. The concentration of ethylene gas was analyzed with a gas chromatograph (GC-8000 series, Fison Instruments, Rodano, Milan, Italy) equipped with FID Detector and a Boraplot Q column (Chrompack capillary column, Varian Inc., Walnut Creek, California, USA). At 14dpi, nematodes inside roots were counted. The whole experiment was performed two times, with ten individual plants in each replicate.

### Microscopic observation of giant cells and callose deposition

Microscopic observation of giant cells was performed as described by Ji et al. [23]. The experiment was repeated twice, with 10 galls in each replicate. Each 2-week-old plant was inoculated with 100 J2 s, and root galls were collected at 7 dpi. After fixation in 1× PIPES buffer overnight, root galls were dehydrated in several ethanol dilutions and embedded with Technovit 7100 for 2 weeks. The embedded gall tissues were sectioned into 10-μm slices with a CryoStar NX50 Cryostat (Thermo Fisher Scientific, MA, USA) and stained with 0.05% toluidine blue for 5 min. Microscopic observations were performed using a IX83 research inverted microscope (Olympus Optical Company, Tokyo, Japan) at 40 magnification.

Callose deposition was examined according to Millet et al. [33]. Rice plants were maintained in 0.04% Si or in control conditions for 2 weeks and inoculated with 100 J2 s each. Ten root galls from each treatment were fixed in an ethanol acetic acid solution overnight and then dehydrated in ethanol dilutions. The root galls were stained with 0.01% aniline blue solution. Callose deposition was examined under UV light using an Eclipse Ti epifluorescence microscope (Nikon Tec. Corporation, Tokyo, Japan) and quantified using ImageJ software.

### Quantification of H_2_O_2_, lignin and total soluble phenolics

Each two-week-old rice plant was maintained in SAP with either 0.04% Si addition or a mock solution and inoculated with 100 J2 s. Before inoculation and at several time points after inoculation with *M. graminicola*, root samples were collected for H_2_O_2_, lignin and total soluble phenolics quantification.

The accumulation of H_2_O_2_ *in planta* was determined using the trichloroacetic acid (TCA) method as described in Velikova et al. [51]. At 6, 24 and 72 hpi, 0.1 g fresh root sample was collected from a pool of six plants and processed as described in Ji et al. [24]. The experiment was performed twice, with four replicates each.

Lignin quantification was performed using the acetyl bromide (AcBr) method as described by Vanholme et al. [49]. At 24 and 48 hpi, fresh roots from six individual plants were collected and dried in a speedvac at − 20 °C for 3 d. Dried roots were ground and extracted in a sequence of water, ethanol, chloroform and acetone. Lignin absorbance was measured at 280 nm and calculated as described in Vega-Sánchez et al. [50]. Each experiment was performed twice, with four replicates each.

Total soluble phenolics were quantified as described by Rodrigues et al. [39]. At 24 and 48 hpi, roots were collected and freeze dried for 3 d. Dried roots were ground and extracted in a sequence of methanol, folin-phenol and sodium carbonate. The absorbance of samples was measured at 725 nm and calculated as described in Zieslin and Ben-Zaken [57]. The experiment was conducted twice, with three replicates per treatment.

To further investigate the role of the lignin synthesis-related phenylpropanoid pathway in Si-induced defense against *M. graminicola*, 100 μM of L-2-aminooxy-3-phenylpropionic acid (AOPP), an inhibitor of phenylpropanoid biosynthesis, was applied to the roots in combination with or without 0.04% Si 24 h before nematode inoculation. Then, the plants were inoculated with 100 J2 s each and investigated at 14 dpi as described above.

### Transcript analysis by qRT-PCR

To analyze the transcript levels of different plant defense-related genes, RNA was extracted from root samples of six individual plants each with an RNeasy Plant Mini kit, and cDNA was synthesized using the SuperScript® II Reverse Transcriptase Kit (Invitrogen, Shanghai, China). The primer sequences used for qRT-PCR analysis of defense-related genes and internal reference genes are listed in Table 1. All of the qRT-PCR analyses were performed with a 7500-fast real time PCR system (Thermo Fisher Scientific, Beijing China) in triplicate with two independent biological replicates as described in Huang et al. [21]. The relative expression level of defense-related genes is shown as the fold change compared with that of the non-amended non-inoculated control plants, which was set at 1.

**Table 1:** List of qRT-PCR primers with GenBank accession/locus numbers (MSU7.0) of the reference and target genes

Genes	GenBank accession or locus number	Primer sequences (5′ → 3′)	Function
*OsACS1*	AK071011	F: GATGGTCTCGGATGATCACA	ET biosynthesis
		R: GTCGGGGGAAAACTGAAAAT	
*OsACO7*	LOC_Os01g39860	F: GGACTACTACCAGGGCACCA	ET biosynthesis
		R: GATTAGCGCACGCGATTTTA	
*OsEIN2*	LOC_Os07g06130	F: TAGGGGGACTTTGACCATTG	ET signaling
		R: TGGAAGGGACCAGAAGTGTT	
*OsEBP89*	LOC_Os03g08460	F: TGACGATCTTGCTGAACTGAA	ET response
		R: CAATCCCACAAACTTTACACA	
*OsERF70*	AF193803.1	F: ACCTTGGGGGTAGCATATCG	ET response
		R: AGGGAACAGGTCCAATCACC	
*OsERF1*	LOC_Os04g46220	F: GAGTCGTCCTTCTCCTCCTC	ET response
		R: CCTCTCTTTCTCCGTTTCG	
*OsC4H*	NM_001061725	F: CAGACTGGTGAGATCCGGTG	Phenylpropanoid biosynthesis
		R: TTCCCCATTCGATCGACCAC	
*OsCAD6*	NM_001058825	F: TCGGTAAGAGGACGGTGAGT	Phenylpropanoid biosynthesis
		R: TGTCGATGTCCCAGGTGATG	
*OsRbohB*	NM001049555.1	F: CTGGACAGGACCAAGAGCAG	H2O2 production
		R: ATCTTGAACGGAGCAGCACA	
*OsEif5C*	SM00515	F: CACGTTACGGTGACACCTTTT	Reference gene
		R: GACGCTCTCCTTCTTCCTCAG	
*OsEXP*	LOC_Os03g27010	F: TGTGAGCAGCTTCTCGTTTG	Reference gene
		R: TGTTGTTGCCTGTGAGATCG	
*OsEXPnarsai*	LOC_Os07g02340.1	F: AGGAACATGGAGAAGAACAAGG	Reference gene
		R: CAGAGGTGGTGCAGATGAAA	

### Statistical analysis

The means and standard errors of the data were subjected to statistical analysis using SAS software ersion 8.0 (SAS Institute, Cary, NC). Significant differences (*P* ≤ 0.05) between the treatments were determined according to Duncan’s multiple range test.

## Abbreviations

DPI: Days post inoculation
ET: Ethylene
H_2_O_2_: Hydrogen peroxide
HPI: Hours post inoculation
IR: Induced resistance
JA: Jasmonic acid
PAL: Phenylalanine
POX: Peroxidase
PPO: Polyphenol oxidase
PR: Pathogenesis related
PVC: Polyvinyl chloride
RKN: Root-knot nematode
SA: Salicylic acid
SAP: Synthetic absorbent polymer
SAR: Systemic acquired resistance
